# Comparative Analysis of Chemical Composition, Antioxidant Activity and Quantitative Characterization of Some Phenolic Compounds in Selected Herbs and Spices in Different Solvent Extraction Systems

**DOI:** 10.3390/molecules23020402

**Published:** 2018-02-13

**Authors:** Shabnam Sepahpour, Jinap Selamat, Mohd Yazid Abdul Manap, Alfi Khatib, Ahmad Faizal Abdull Razis

**Affiliations:** 1 Department of Food Science, Faculty of Food Science and Technology, Universiti Putra Malaysia, 43400 Serdang, Selangor, Malaysia; Sh.sepahpour@yahoo.com (S.S.); madfaizal@upm.edu.my (A.F.A.R.); 2 Food Safety and Food Integrity (FOSFI), Institute of Tropical Agriculture and Food Security, Universiti Putra Malaysia, 43400 Serdang, Selangor, Malaysia; 3 Department of Food Technology, Faculty of Food Science and Technology, Universiti Putra Malaysia, 43400 Serdang, Selangor, Malaysia; myazid@upm.edu.my; 4 Halal Products Research Institute, Universiti Putra Malaysia, 43400 Serdang, Selangor, Malaysia; 5 Department of Pharmaceutical Chemistry, Faculty of Pharmacy, International Islamic Universiti Malaysia, 25200 Kuantan, Pahang, Malaysia; alfikhatib@iium.edu.my; 6 Laboratory of Molecular Biomedicine, Institute of Bioscience, Universiti Putra Malaysia, 43400 Serdang, Selangor, Malaysia

**Keywords:** turmeric, curry leaf, torch ginger, lemon grass, solvent extraction, total phenolic content, total flavonoid content, antioxidant activity

## Abstract

This study evaluated the efficacy of various organic solvents (80% acetone, 80% ethanol, 80% methanol) and distilled water for extracting antioxidant phenolic compounds from turmeric, curry leaf, torch ginger and lemon grass extracts. They were analyzed regarding the total phenol and flavonoid contents, antioxidant activity and concentration of some phenolic compounds. Antioxidant activity was determined by the 2,2-diphenyl-1-picrylhydrazyl (DPPH) free radical scavenging assay and the ferric reducing antioxidant power (FRAP) assay. Quantification of phenolic compounds was carried out using high-performance liquid chromatography (HPLC). All the extracts possessed antioxidant activity, however, the different solvents showed different efficiencies in the extraction of phenolic compounds. Turmeric showed the highest DPPH values (67.83–13.78%) and FRAP (84.9–2.3 mg quercetin/g freeze-dried crude extract), followed by curry leaf, torch ginger and lemon grass. While 80% acetone was shown to be the most efficient solvent for the extraction of total phenolic compounds from turmeric, torch ginger and lemon grass (221.68, 98.10 and 28.19 mg GA/g freeze dried crude extract, respectively), for the recovery of phenolic compounds from curry leaf (92.23 mg GA/g freeze-dried crude extract), 80% ethanol was the most appropriate solvent. Results of HPLC revealed that the amount of phenolic compounds varied depending on the types of solvents used.

## 1. Introduction

In recent years, natural phytochemicals existing in herbs and spices have been widely used to cure, inhibit or reduce the risk of human diseases [1,2]. Phytochemicals have potential health benefits due to their antioxidant activities and inhibitive effects against oxidative damage that has been implicated in a number of illnesses, specifically cancer and cardiovascular diseases [3].

Among the phytochemical substances, phenolic compounds, including phenolic acids and flavonoids, are the major groups of natural components in plants which have received increasing interest over the last decades due to free radical scavenging properties. These bioactive compounds vary in type, number and position of functional groups, resulting in variations in chemical properties which can influence the solubility of these compounds in different solutions [4]. It is also reported that the profile of phenolic compounds extracted from material is dependent on the polarity of the solvents used for extraction [5,6,7]. Hence, selecting the best solvent is a key factor which impacts the quality and quantity of extracted phenolic compounds.

Spices and herbs, used in food to improve color and flavor, are well known for their antioxidant properties. Turmeric, curry leaf, lemon grass and torch ginger are spices and herbs which are widely used as essential ingredients in preparation of different cuisines and medicinal treatments in South and Southeast Asia. It has been reported that they are rich in antioxidant components, namely polyphenols, which are responsible for their medicinal properties [8,9,10,11]. Because the chemical structure of the constitution of the samples, and the selection of appropriate solvents determine the type of polyphenols eluted in solvents, a general extraction technique cannot be recommended for the recovery of all types of phenolic compounds from all plant sources. Therefore, the main objectives of this study were to compare some aqueous organic solvents and pure water for their ability to extract phenolic compounds from turmeric (*Curcuma longa*), curry leaf (*Murraya koenigii*), torch ginger (*Etlingera elatior*) and lemon grass (*Cymbopogon citratus*), and to quantify some of the phenolic compounds of turmeric (curcumin, desmethoxycurcumin and bis-desmethoxycurcumin), curry leaf (rutin, quercetin-3-glycoside, myrecitin, quercetin), torch ginger (chlorogenic acid) and lemon grass (caffeic acid, *p*-coumaric acid, luteolin-7-*o*-glycoside) in different solvent extraction systems in order to propose the most efficient solvent for extractions of phytochemicals from these plants.

## 2. Results and Discussion

### 2.1. Total Phenolic Compounds

Table 1 presents the amount of total phenol content from each sample extracted by different solvents. The quantity of total phenol from the extracts indicates very wide variation. Acetone extract of turmeric exhibited the highest quantity of total phenol content (221.7 mg gallic acid equivalent (GAE)/g of freeze-dried crude extract (CE) while water extract of lemon grass demonstrated the lowest amount to total phenolic compounds (1.2 mg GAE/g CE). The results revealed that the most efficient aqueous solvent for extraction of total phenolic content (TPC) for turmeric is 80% acetone, followed by 80% ethanol and 80% methanol. The higher total phenolic content in 80% acetone solvent extraction of turmeric may be due to the fact that major phenolic compounds of turmeric (curcuminoids) are made of a long nonpolar chain of carbon–carbon covalent bonds with a phenolic group attached to the two ends [12]. The structure allows them to dissolve most freely in acetone with lower polarity (as obtained from this study), followed by ethanol, methanol and only partially in water due to water’s high polarity.

The results showed that water yielded the lowest quantity of TPC for all samples. The highest value of TPC for turmeric, torch ginger and lemon grass were observed in 80% acetone extraction, whereas 80% ethanolic extraction showed the highest TPC for the curry leaf. Several studies have illustrated that 80% acetone was a more effective solvent for the extraction of polyphenols. According to Sulaiman, et al. [13] 70% acetone was considered to be the most efficient solvent for extracting TPC of 24 vegetables from 37 selected plants. The high efficiency of acetone to extract TPC from samples may be due to its ability to prevent the protein–polyphenol binding, which is insoluble complex, through solvent extraction [14]. It has been postulated that acetone is able to inhibit the formation of the protein–polyphenol complex during extraction, or to break down the interaction between the functional group of polyphenols (–OH) and the carboxyl group of proteins [7]. Regardless of the type of solvents used, turmeric exhibited the highest TPC value followed by curry leaf, torch ginger and lemon grass. Among samples obtained through water extraction, curry leaf and lemon grass held the highest and lowest TPC values: 34.7 and 1.2 mg GAE/g CE, respectively.

### 2.2. Total Flavonoid Content

**Figure 1:**
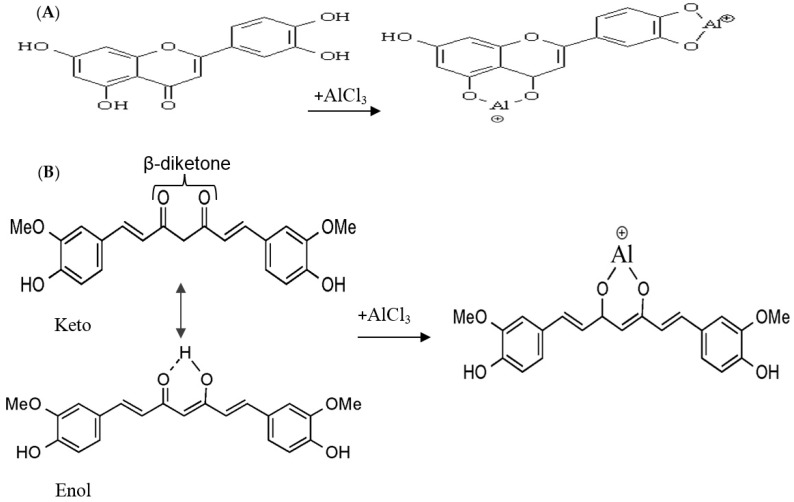
Formation of flavonoid complex with aluminum (AlCl_3_) (**A**); formation of curcumin with (AlCl_3_) (**B**).

The total flavonoid content (TFC) of samples extracted by different organic solvents was reported in Table 1. Surprisingly, the highest and lowest flavonoid content value belong to turmeric, at 549.2 mg quercetin equivalent/g of freeze-dried crude extract (mg QE/g CE) for 80% acetone extraction and 0.6 mg QE/g CE for water extraction, respectively. Curcuminoids are practically insoluble in water at acidic or natural pH. This could be the explanation for the low level TFC in water extract [15]. Although curcuminoids are not classified as a flavonoid, they act the same way as flavonoid compounds when they react with AlCl_3_ in the TFC method. In fact, curcuminoids are natural diarylheptanoids, which consist of two aromatic o-methoxy phenolic groups linked by a seven-carbon chain (Figure 1B). They exist in enol or keto form; however, the enol configuration is the major component in solution [16]. The principle involved in total flavonoid content is that AlCl_3_ forms acid-stable complexes with the keto groups and either hydroxyl groups of flavonoids (Figure 1A), while it binds to curcuminoids through the β-diketon group [17] (Figure 1B). Therefore, the high concentration of total flavonoid content from turmeric could be linked to the high amount of curcuminoids in turmeric extracts.

Interestingly, a similar trend of TPC was observed for TFC, where 80% acetone extraction of all samples demonstrated higher levels of flavonoid content than other types of solvents except for curry leaf, where its flavonoids showed higher solubility in 80% ethanol and 80% methanol than in 80% acetone. This may be due to the fact that the majority of the flavonoids of curry leaf are conjugated with different types of sugars, such as quercetin-3-glucoside, quercetin-*O*-pentohexoside, kaempferol-*O*-glucoside and quercetin-3-*O*-rutinoside [10]. The chemical structure of these conjugated compounds makes them more soluble in alcoholic solvent compared to 80% acetone. The higher solubility of sugars can be associated with the dielectric constant of ethanol and methanol (24.55 and 32.78, respectively, at 25 °C), which is more similar to water (78.54 at 25 °C) than acetone is (20.7 at 25 °C) [18]. In fact, the dielectric constant is a good indicator of solvent polarity. Hence, it is obvious that a water–alcohol solvent mix is much more polar than a water–acetone solvent mix. Consequently, polar constituents can dissolve easily in such a solvent. Singh et al. [10], quantifying 10 different flavonoids of curry leaf in various aqueous solvents reported that 6 of the 10 curry leaf flavonoids showed more recovery in 80% ethanol than 80% acetone.

In 80% acetone extractions of samples, turmeric possessed the highest total flavonoid content (549.2) followed by curry leaf (47.6 mg QE/g CE), torch ginger (38.1 mg QE/g CE) and lemon grass (14.8 mg QE/g CE). The same trend was evident for 80% methanolic and ethanolic extractions of samples, whereas in water extraction, torch ginger showed the highest TFC (12.3 mg QE/g CE) followed by lemon grass (3.7 mg QE/g CE), curry leaf (2.8 mg QE/g CE) and turmeric (0.6 mg QE/g CE).

### 2.3. Antioxidant Properties

#### 2.3.1. 2,2-Diphenyl-1-picrylhydrazyl (DPPH) Free Radical Scavenging

The free radical scavenging ability of samples was investigated using DPPH assay. In this assay, the initial purple color of the DPPH radical became yellow due to the presence of hydrogen or electrons provided by antioxidant agents of the samples. The results demonstrated that the TPC was significantly (*p* < 0.001) influenced by the type of solvents and plant material. Table 2 indicates that all different sample extracts had antioxidant capacity which varied by the polarity of the solvents. In the 80% acetone extraction, turmeric showed the greatest free radical scavenging activity, at 67.8%, whereas lemon grass had the least DPPH radical scavenging activity, at 11.8%. Results of the DPPH assay for turmeric, curry leaf, lemon grass and torch ginger in various solvent extractions showed similar trends as TPC and TFC, where organic solvents were far more effective than pure water for extracting compounds such as polyphenols, which are significantly linked to the extract’s antioxidant properties. Similar results have been reported for other raw materials such as black tea [19], grape [20], and murta leaves [21], since their antioxidant compounds had much higher yield in organic solvents than in pure water. According to some studies, pure water is not an efficient solvent to extract polyphenols because these compounds are more soluble in solvents less polar than water [5,18,22,23]. The trend of effective solvents for curry leaf was different that of the other samples, where 80% methanolic and ethanolic extracts of curry leaf were more powerful than the 80% acetone extract when measured for the inhibition of the radical activity of DPPH in other sample extracts. Therefore, this result showed that alcoholic solvents are more efficient solvents than acetone and water for the extraction of phenolic compounds from curry leaf. Similar to the results reported by Koffi, et al. [24], which showed that ethanol was the most efficient solvent for the extraction of phenolic compounds in Ivorian plants. Moreover, a research conducted by Anokwuru, et al. [25] indicated that the methanol extract of hibiscus recorded the highest inhibition of DPPH.

#### 2.3.2. Ferric Reducing Antioxidant Power

The reducing power of plant extracts, which is associated with antioxidant activity, was measured using the ferric reducing antioxidant power (FRAP) assay. Samples which possessed antioxidant compounds were able to reduce Fe(III) in potassium ferricyanide to Fe(II) resulted in changing the solution color from yellow to light green. The results illustrated in Table 2. indicate that there is a considerable variation among and within extracted samples, ranging from 85 mg QE/g CE of turmeric extract with 80% acetone extraction to 0.5 mg QE/g CE of lemon grass extract with water extraction. The results summarized in Table 2 also show that the samples which possessed greater phenolic and flavonoid content showed higher reducing powers. Similar to DPPH, the lowest reducing power was found in all extracts obtained by 100% water (7.4–0.5 mg QE/g CE), demonstrating that the water solubility of active compounds in the samples related to reducing power was very low. This is in accordance with the finding reported by Meneses, et al. [4] and Wang, et al. [26], indicating that aqueous solvents were more efficient than pure water for extracting polyphenols. Table 2 shows that the reducing power of turmeric varied significantly (*p* < 0.001) within different solvent extractions, ranging from 85 to 2.3 mg QE/g CE, whereas, there was no difference in the FRAP value of lemon grass extracted by organic solvents, except for water extraction which showed the lowest FRAP value (0.5 mg QE/g CE). This means that the extractability of compounds with reducing power properties in the water extraction of lemon grass was higher than in other samples. The trend of FRAP was comparable with that obtained by TPC, TFC and DPPH, where turmeric obtained the highest rank followed by curry leaf, torch ginger and lemon grass, which showed the lowest reducing power.

### 2.4. Quantification of Bioactive Compounds of the Samples

**Figure 2:**
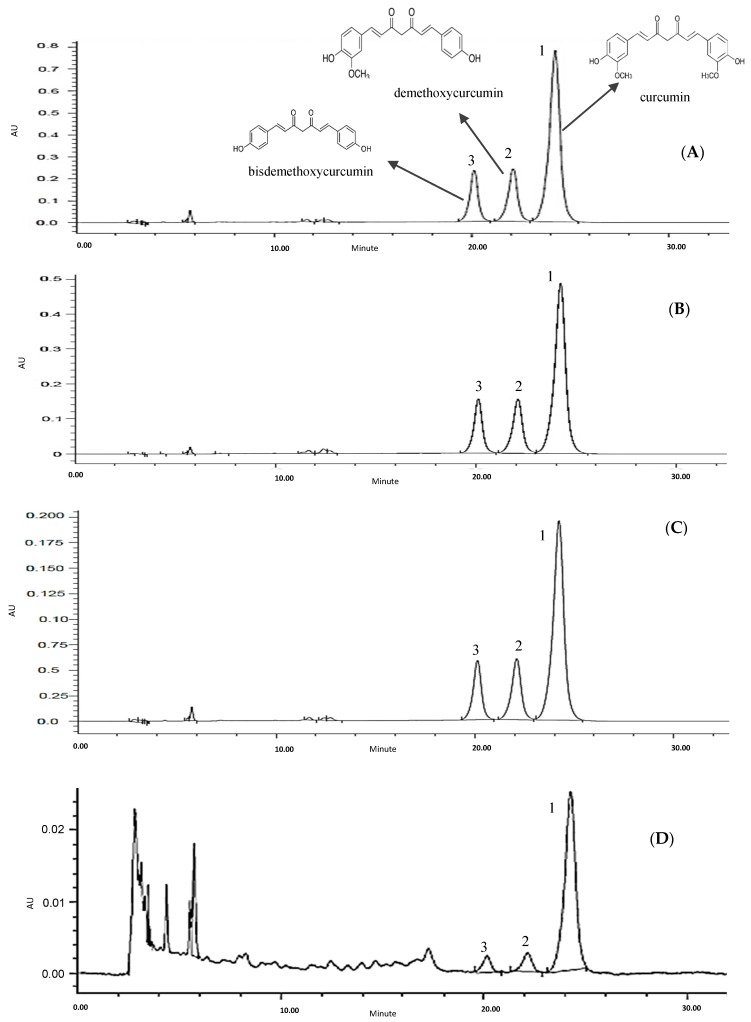
High-performance liquid chromatography (HPLC) chromatograms of turmeric extracted by acetone (**A**); methanol (**B**); ethanol (**C**); and water (**D**).

**Figure 3:**
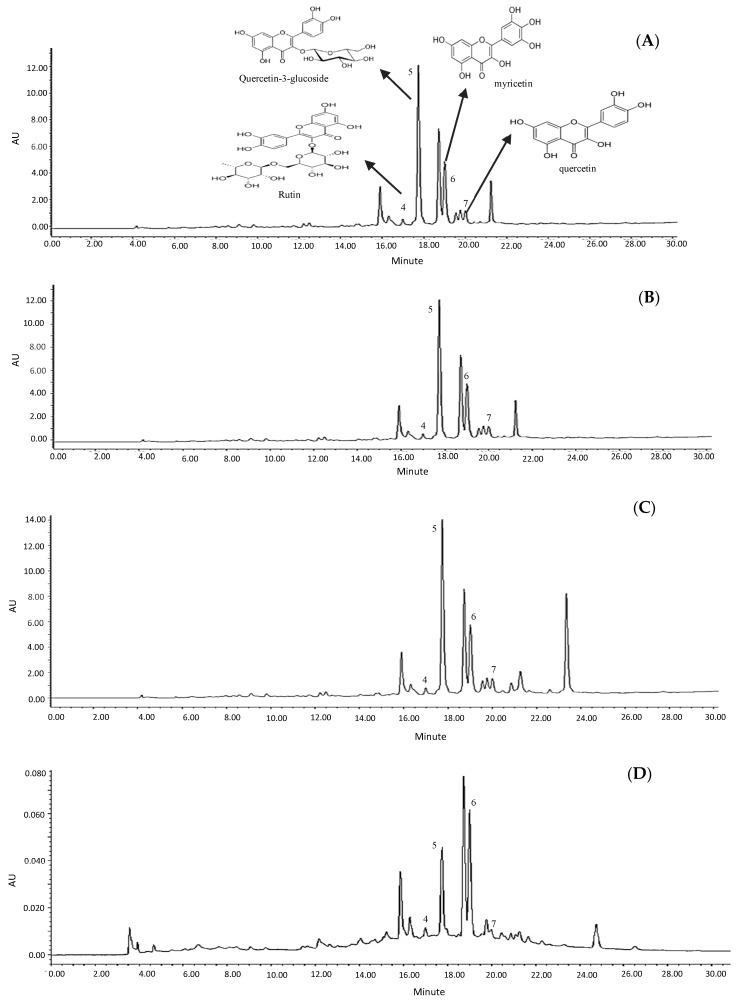
HPLC chromatograms of curry leaf extracted by acetone (**A**); methanol (**B**); ethanol (**C**); and water (**D**).

**Figure 4:**
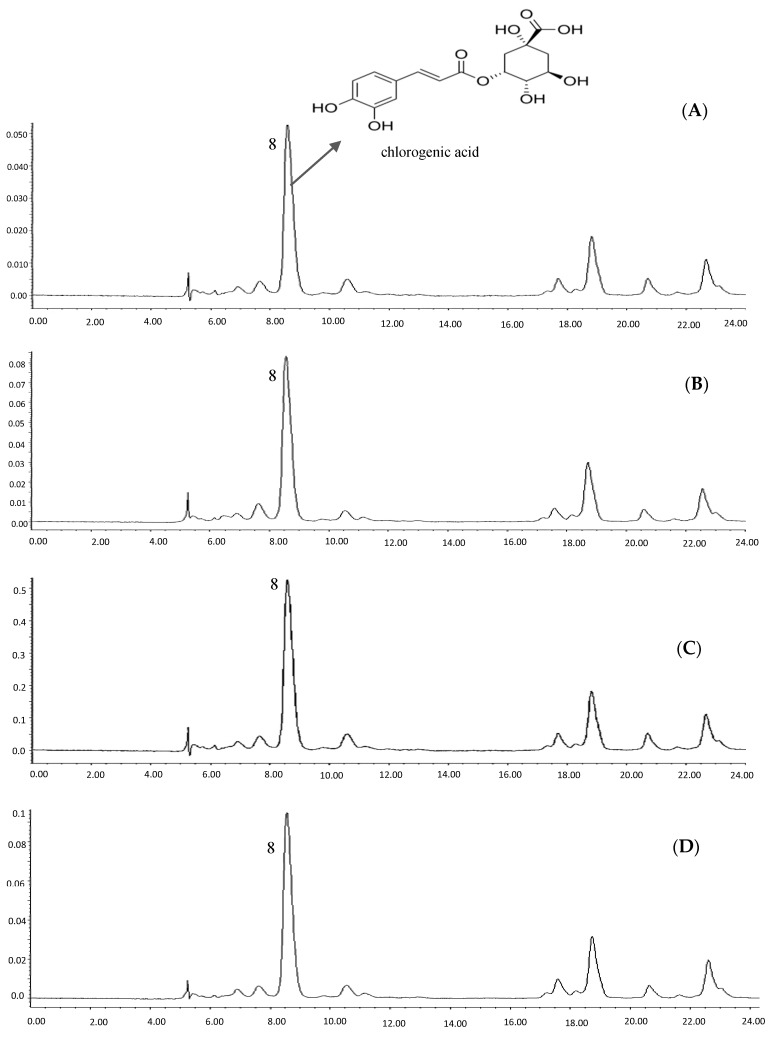
HPLC chromatograms of torch ginger extracted by acetone (**A**); methanol (**B**); ethanol (**C**); and water (**D**).

**Figure 5:**
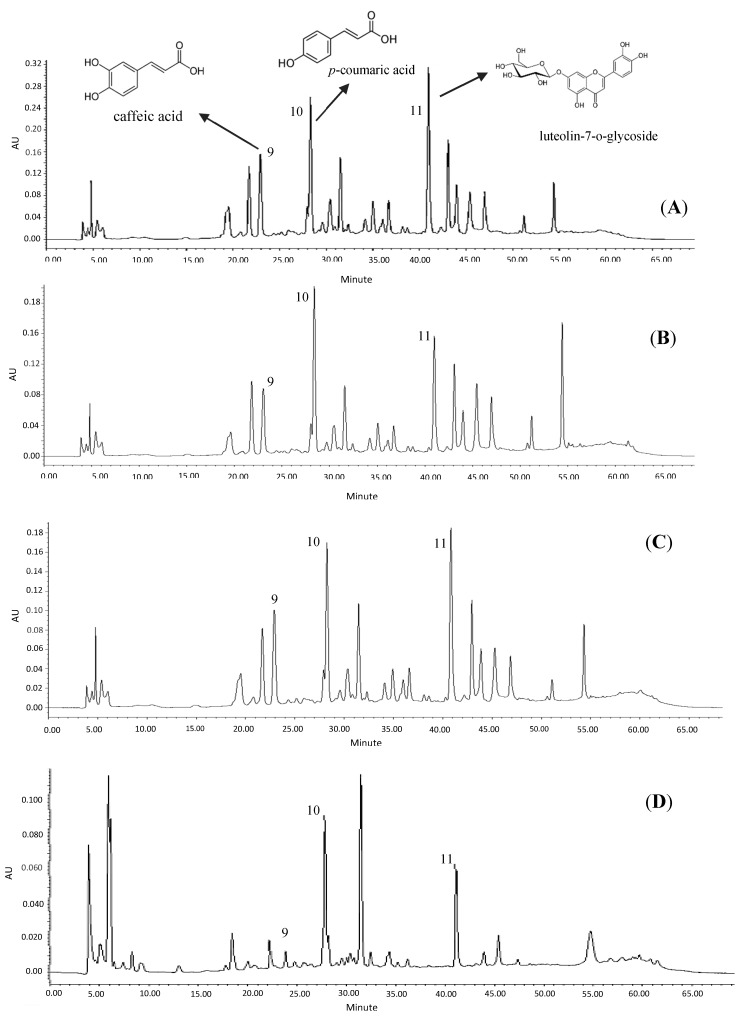
HPLC chromatograms of lemon grass extracted by acetone (**A**); methanol (**B**); ethanol (**C**); and water (**D**).

In this study, the effects of various solvents on the extractability and the quantity of polyphenols from samples were investigated. Since there was a limitation in providing commercial standards, the identification of all peaks in all samples extracts by high-performance liquid chromatography (HPLC) was impossible. Hence, the standards used in this research were selected based on compounds that have been found in the herbs/spices from previous works. Figure 2, Figure 3, Figure 4 and Figure 5 display HPLC profiles of different solvent extractions of turmeric, curry leaf, lemon grass and torch ginger, respectively. According to the retention time, authentic standards and UV spectra, the peaks in these figures were identified as curcumin (1); desmethoxycurcumin (2); bis-desmethoxycurcumin (3); rutin (4); quercetin-3-glycoside (5); myrecitin (6); quercetin (7); chlorogenic acid (8); caffeic acid (9); *p*-coumaric acid (10); luteolin-7-*o*-glycoside (11). Quantification of all of the marked peaks is summarized in Table 3. The HPLC profile of turmeric extraction (Figure 2A–D) revealed that three major bioactive compounds of turmeric (curcuminoids, peaks 1, 2 and 3) could be extracted by all different types of solvents, with significant differences (*p* < 0.05) in their quantity (Figure 2). Acetone (80%) was the most efficient solvent to recover turmeric bioactive compounds, with 510.8, 133.1 and 107.2 (mg/g CE) of turmeric for curcumin, desmethoxycurcumin and bis-desmethoxycurcumin, respectively. Water, as a weak solvent, extracted the lowest value of curcuminoids, ranging from 0. 2 to 0.01 mg/g CE of turmeric. Our results agreed with those of Liu, et al. [27], Maheshwari, et al. [28], and Shaikh, et al. [29], who reported that curcuminoids are weakly soluble in acidic and neutral water. Wichitnithad, et al. [8] quantified the percentages of curcumin, desmethoxycurcumin and bisdesmethoxycurcumin in five commercial turmeric extracts. They reported that curcumin had the highest availability, ranging between 60.19 and 71.50% followed by desmethoxycurcumin at 17.40–27.83% and bisdesmethoxycurcumin at 2.65–4.43% (% *w*/*w*). The differences between the yield of extraction of curcuminiods in our finding and the previous study were likely due to different methods and solvents, different methods of drying samples and different regions where turmeric was harvested. The results of the quantification of turmeric bioactive compounds coincided with TPC, TFC, DPPH and FRAP, which indicates that the proper solvent for turmeric extraction is 80% acetone, followed by 80% ethanol, 80% methanol and water.

Although it has been reported that flavonoids with sugar moieties (flavonoids glycosides) are more water soluble, the concentrations of quercetin-3-glycoside in curry leaf water extract and luteolin-7-*o*-glycoside in lemon grass water extract were far less than those in other solvent extracts. This is because the polarity of organic solvents is less than water, hence, they are able to dissolve the lipid components of the cell membranes and make them more permeable. As a result, higher concentrations of components of plant materials are released to the solvents [30]. This can be seen from Table 3, which shows that the concentration of phytochemicals in water extracts of all the used plant materials were far less than those in other solvent extracts.

The HPLC profile of curry leaf in four different solvent extractions was presented in Figure 3A–D. According to the retention time of authentic standard and UV spectra (366 nm), peaks 4, 5, 6 and 7 were identified as rutin, quercetin-3-glycoside, myricetin and quercetin, respectively. The result of the quantification curry leaf flavonoids in different solvent extractions systems (Table 3) indicated that 80% ethanol was the most efficient solvent for the recovery of quercetin-3-glycoside, myrecitin and quercetin, while 80% acetone extracts showed greater quantities of rutin. Extraction with 80% methanol yielded 0.9, 5.4, 2.4 and 1.4 mg/g of freeze-dried crude extract of rutin, quercetin-3-glycoside, myricetin and quercetin, respectively, which are higher than those obtained in acetone, methanol and water extracts.

Interestingly, a new peak in retention time of 23.48 min was observed in 80% ethanol extract of curry leaf while it was not seen in 80% acetone, 80% methanol and water extracts. Therefore, the high level of total flavonoid content and antioxidant activity of ethanol extracts might be attributed to this unknown compound which is only extracted by 80% ethanol.

Figure 4A–D show the HPLC results of different solvent extractions of torch ginger. A typical peak at retention time around 8.6 min was allocated to chlorogenic acid, which is identified in all types of solvent extracts by comparing its retention time with standard and UV spectra (265 nm). Quantification was measured based on the calibration curve made by plotting the peak area values versus chlorogenic acid standard concentrations. The quantification of chlorogenic acid was presented in Table 3. The highest amount of this compound was extracted by 80% ethanol, at 21.8 mg/g CE, followed by 80% methanol (18.7 mg/g CE), water (13.4 mg/g CE) and 80% acetone (11.5 mg/g CE). Quantification of chlorogenic acid among 24 vegetables showed that it was the abundant phenolic acid in 18 of 24 vegetables studied [1]. The amount of this compound in torch ginger was quantified at 14.06 mg/100 g fresh weight (fw), which was far lower than what we recorded. The substantial difference between our results and those of Andarwulan et al. [1] were likely due to different solvent extraction systems whereby they used 5% HCl as an extraction solvent. Moreover, they reported the quantity based on the fresh weight of the samples, while the present study reported all values based on the weight of freeze-dried crude extract.

The HPLC profile of lemon grass is displayed in Figure 5A–D. In all different solvent extractions, peaks 9, 10 and 11 were verified upon comparing their retention time with standards and UV spectra (280 nm), as caffeic acid, *p*-coumaric acid and luteolin-7-*o*-glycoside. They were quantified using linear calibration curves of their commercial standards. According to the results presented in Table 3, 80% acetone gave the highest yield of caffeic acid, *p*-coumaric acid and luteolin-7-*o*-glycoside 0.3, 0.5 and 0.6 mg/g CE, respectively. The HPLC profiles of water extraction confirmed that the lower values of TPC, TFC, DPPH and FRAP in water extractions were attributed to the lower quantity and quality of bioactive compounds extracted by water.

## 3. Materials and Methods

### 3.1. Chemical Reagents

Solvents were purchased from Merck (Darmstadt, Germany). All chemical standards including curcumin, demethoxycurcumin, bisdemethoxycurcumin, rutin, quercetin-3-glycoside, myrecitin, quercetin, chlorogenic acid, caffeic acid, *p*-coumaric acid, luteolin-7-*o*-glycoside were purchased from Sigma–Aldrich (St. Louis, MO, USA). Folin–Ciocalteau reagent, 1,1-diphenyl-2-picrylhydrazyl (DPPH), ferric chloride, Na_2_CO_3_, NaNO_2_, AlCl_3_, KH_2_PO_4_, potassium ferric cyanide, and trichloroacetic acid (TCA) were obtained from Merck (Darmstadt, Germany).

### 3.2. Plant Material

All herbs and spices, rhizome of turmeric, inflorescence of torch ginger, leaves of lemon grass and leaves of curry leaf were freshly purchased from a local market in Selangor, Malaysia. The samples were cleaned and washed thoroughly under running water. Then, the excess water was drained and the samples were freeze-dried. Then they were ground to a fine powder using a mechanical kitchen blender (Model DPA1, Tefal, Rumilly, France) and extracted with 80% methanol, 80% ethanol, 80% acetone and water for 1 h using a magnetic stirrer at room temperature. Extraction was repeated three times. The ratio of sample to solvent was 1:10 (*w*/*v*). The extracts were then filtered through Whatman No. 1 filter paper (Whatman International Ltd., Maidston, UK) and concentrated with a vacuum rotary evaporator (Buchi, Rotavapor R-210, Flawil, Switzerland). The residue was freeze-dried and kept at −18 °C prior to further analysis.

### 3.3. Determination of Total Phenolic Content

The TPC of the herb/spice extracts was quantified using the Folin–Ciocalteu (FC) assay [31]. An aliquot (1 mL) of diluted (0.8 mg/mL) extract solution was dissolved with 5 mL of FC reagent, which was pre-diluted 10 times with distilled water. Mixture was kept for 5 min at room temperature, followed by adding 4 mL of sodium carbonate solution (Na_2_CO_3_, 7.5% *w*/*v*). The solutions were shaken and stood for 1 h at room temperature. The absorbance of the resulting blue color was recorded at 765 nm using Genesys 10S UV-Vis spectrophotometer (Thermo Fisher Scientific, Waltham, MA, USA). 40, 50, 60, 70 and 80 µg/mL Gallic acid dissolved in methanol was used as the standard for making a calibration curve (*R*^2^ = 0.996) to determine the total phenol content. Results were expressed as mg Gallic acid equivalent/g of freeze-dried crude extract (mg GAE/g CE).

### 3.4. Determination of Total Flavonoid Content

The colorimetric assay reported by Wijekoon, et al. [9] was employed to measure the TFC in extracts. A volume of 500 µL of diluted extract (0.8 mg/mL) was dissolved in 2.5 mL of distilled water followed by the addition of 150 µL of 5% (*w*/*v*) sodium nitrite (NaNO_2_) to the solution. After 5 min, 300 µL of 10% (*w*/*v*) aluminum chloride (AlCl_3_) was mixed and the solution was allowed to stand for 5 min before the addition of 1 mL of 1 M NaOH solution. The mixture was diluted with 550 µL of distilled water and shaken vigorously. The solution absorbance was read at 510 nm using Genesys 10S UV-Vis spectrophotometer (Thermo Fisher Scientific, Waltham, MA, USA). Quercetin was used as the standard at concentrations 100, 150, 200, 250 and 300 µg/mL, (*R*^2^ = 0.993). The results were reported as mg quercetin equivalent/g of freeze-dried crude extract (mg QE/g CE).

### 3.5. Ferric Reducing Antioxidant Power Assay

The FRAP was determined according to the modified method described by Shon, et al. [32]. In this assay, the reducing power was recorded by measuring the reduction of Fe^+3^ into Fe^+2^. An aliquot (1 mL) of methanolic extract solution was diluted in 2.5 mL of 20 M phosphate buffer (pH: 6.6), 2.5 mL 1% (*w*/*v*) potassium ferricyanide followed by incubation at 50 °C for 30 min. After incubation, 2.5 mL of trichloroacetic acid 10% was added to the solution and centrifuged (Sigma 3–18 K, Sartorius, Gettingen, Germany) at 650 g for 10 min. The supernatant (5 mL) was taken and mixed with distilled water (5 mL), followed by 500 µL ferric chloride solution (1% *w*/*v*) and mixed thoroughly. The solution was incubated at ambient temperature for 10 min. The absorbance was recorded at 700 nm using Genesys 10-S UV-Vis spectrophotometer (Thermo Fisher Scientific, Waltham, MA, USA) and expressed as milligram quercetin equivalent/g of freeze-dried crude extract (mg QE/g CE).

### 3.6. DPPH Free Radical-Scavenging Assay

The antioxidant capacity of the extracts was measured in terms of the method described by Álvarez-Casas, et al. [33] using the 1,1-diphenyl-2-picrylhydrazyl (DPPH) radical scavenging assay. An aliquot (100 µL) of extracts (0.8 mg/mL) was mixed with 3.9 mL of 0.1 mM methanolic DPPH solution. The mixture was thoroughly mixed and allowed to stand in the dark for 30 min at room temperature. The absorbance of the solution was read at 517 nm. Results were expressed as the percentage of inhibition of the DPPH radical which was calculated according to the following equation:

(1) $$\%\;\mathrm{inhibition}=\frac{A\;\mathrm{control}-A\;\mathrm{sample}}{A\;\mathrm{control}}\times 100$$

where A control is the absorbance of the DPPH without plant extracts, and A sample is the absorbance of the DPPH after adding extracts. All tests were repeated in triplicate.

### 3.7. Identification and Quantification of Bioactive Compounds

The HPLC separation system was applied to determine the amount of some targeted phenolic compounds of each extract. An Aliquot of 20 µL of the extracts was injected into Waters 600 HPLC system (Milford, MA, USA) with a UV-diode array detector system equipped by Hypercil Gold column C18 (5 µm, 250 × 4.6 mm, Thermo Fisher Scientific, Waltham, MA, USA). Various gradient programs were performed for different samples in order to achieve the optimum efficiency of chromatographic separation for each extract. The quantification of turmeric active compounds was carried out using an isocratic method described by Wichitnithad, et al. [8] with the following small modification: Isocratic acetonitrile, 2% acetic acid 40:60 at 1 mL/min of flow rate for 30 min was used with detection of flavonoids at 425 nm. The column temperature was set at 33 °C. For torch ginger and lemon grass, the same mobile phases, A: 0.2% aqueous formic acid and B: methanol, with different gradients at a flow rate of 0.8 mL min^−1^ were used. Column temperature was set at 24 °C. The gradient HPLC for lemon grass described by Figueirinha, et al. [11] started with 95–85% A (0–10 min), 85–70% A (10–15 min), 70–65% B (15–25 min), 65–50% A (25–35 min), 50–20% A (35–40 min), followed by isocratic 20% A for 20 min. Chromatographic profiles were acquired in the wavelength 280 nm. For torch ginger, a modified gradient was used: 75–60% A (0–15 min), 60–80% A (15–25 min), 80–90% A (25–30 min), 90–75% A (30–40 min). The compound was monitored at 265 nm. A gradient chromatographic separation was performed [10] in order to determine the curry leaf bioactive compounds at ambient temperature using a mobile phase of solvent A: 10% methanol at pH 3.5 with 0.01% formic acid, and solvent B: methanol, water, acetonitrile (20:20:60) at pH 3.5 with 0.01% formic acid, with a constant flow rate of 1 mL/min, ambient temperature for the column, and a detection wavelength of 366 nm. The gradient program was: 0–5 min 100% A, 5–10 min 85% A, 10–20 min 80% A, 20–25 min 75% A, 25–27 min 73% A, 27–30 min 60% A, 30–35 min 50% A, A: 35–40 min 10% A, and returned to 100% A for 20 min.

### 3.8. Statistical Analysis

Minitab software for windows (Version 16, Minitab Inc., State College, PA, USA) was employed to analyze data. All data were obtained in triplicate and expressed as the mean value ± standard deviation. Analysis of variance (ANOVA) followed by Tukey’s honestly significant different (HSD) method were used to seek significant differences between means. Differences were specified as statistically significant with *p*-values below 0.05 (*p* < 0.05).

## 4. Conclusions

This study investigated the of effect different organic solvents on the phenolic content of extracts from turmeric, curry leaf, torch ginger lemon grass. All spices and herbs utilized in this study possess antioxidant properties, with turmeric extract exhibiting higher contents of antioxidant constituents, which is responsible for its stronger antioxidant capacity than other samples. Among the herbs/spices, turmeric showed the highest TPC, TFC, and DPPH antioxidant activity (47.35%), and FRAP, followed by curry leaf, torch ginger and lemon grass. Acetone (80%) was demonstrated to be the best solvent for the extraction of turmeric antioxidant compounds, whereas, for curry leaf, torch ginger and lemon grass, 80% alcoholic solvents were superior to 80% acetone. Antioxidant activity of the all samples showed the lowest values in water extraction compared to organic solvents. Generally, high TPC and TFC correlated with high DPPH and FRAP values, indicating that polyphenols were mainly responsible for the antioxidant activities of the extracts. This study also suggests that the extraction solvent can affect the phytochemical profile and the antioxidant activity of these extracts.

## Figures and Tables

**Table 1:** Total phenolic and total flavonoid content of samples obtained from different solvent extraction systems.

Plant Sources	TPC (mg GAE/g Freeze-Dried Crude Extract)				TFC (mg QE/g Freeze-Dried Crude Extract)			
	80% Acetone	80% Ethanol	80% Methanol	Water	80% Acetone	80% Ethanol	80% Methanol	Water
Turmeric	221.7 ± 0.9 ^A,a^	172.1 ± 1.4 ^B,a^	90.1 ± 2.0 ^C,a^	3.8 ± 0.1 ^D,c^	549.2 ± 4.5 ^A,a^	380.7 ± 5.5 ^B,a^	133.0 ± 3.9 ^C,a^	0.6 ± 0.1 ^D,d^
Curry leaf	83.8 ± 0.9 ^B,b^	92.2 ± 1.7 ^A,b^	86.7 ± 2.0 ^B,a^	34.7 ± 1.0 ^C,a^	47.6 ± 1.2 ^C,b^	144.5 ± 2.9 ^A,b^	83.4 ± 3.2 ^B,b^	2.8 ± 0.1 ^D,c^
Torch ginger	97.1 ± 4.8 ^A,b^	80.4 ± 2.1 ^B,c^	88.4 ± 3.5 ^B,a^	16.4 ± 0.2 ^C,b^	38.1 ± 1.4 ^A,c^	39.7 ± 3.3 ^A,c^	36.7 ± 0.96 ^A,c^	12.3 ± 0.1 ^B,a^
Lemon grass	28.2 ± 0.6 ^A,c^	25.3 ± 0.7 ^A,d^	22.1 ± 0.4 ^B,b^	1.2 ± 0.1 ^C,d^	14.8 ± 0.5 ^A,d^	14.3 ± 0.1 ^A,d^	11.7 ± 1.1 ^B,d^	3.7 ± 0.1 ^C,b^

Means with different small letters (a–d) in the same column are significantly different. Means with different capital letters (A–D) in the same row are significantly different. Number of replicate = 3. Abbreviations: TPC, total phenolic content; TFC, total flavonoid content; QE, quercetin equivalent.

**Table 2:** DPPH and FRAP values of samples extracts obtained from different solvent extraction systems.

Plant Sources	DPPH (%)				FRAP (mg QE/g Freeze-Dried Sample Extract)			
	80% Acetone	80% Ethanol	80% Methanol	Water	80% Acetone	80% Ethanol	80% Methanol	Water
Turmeric	67.8 ± 4.0.97 ^A,a^	47.4 ± 2.6 ^B,a^	27.8 ± 0.9 ^C,b^	13.8 ± 3.4 ^D,a^	85.0 ± 1.3 ^A,a^	55.8 ± 0.4 ^B,a^	25.4 ± 1.0 ^C,c^	2.3 ± 0.2 ^D,c^
Curry leaf	29.8 ± 1.1 ^B,b^	41.74 ± 0.9 ^A,b^	31.5 ± 0.5 ^B,a^	8.2 ± 2.1 ^C,a,b^	42.3 ± 2.7 ^B,b^	52.4 ± 2.4 ^A,b^	44.4 ± 1.7 ^B,a^	3.2 ± 0.2 ^C,b^
Torch ginger	32.7 ± 5.2 ^A,b^	27.0 ± 1.8 ^A,c^	28.4 ± 4.2 ^A,b^	11.5 ± 1.8 ^B,a,b^	36.1 ± 3.2 ^B,c^	40.0 ± 1.4 ^A,B,b^	40.9 ± 0.6 ^A,b^	7.4 ± 0.4 ^C,a^
Lemon grass	11.8 ± 0.8 ^A,c^	10.4 ± 1.1 ^A,d^	11.1 ± 0.7 ^A,c^	7.2 ± 0.4 ^B,b^	10.3 ± 1.4 ^A,d^	8.4 ± 1.0 ^A,c^	9.8 ± 1.5 ^A,d^	0.5 ± 0.1 ^B,d^

Means with different small letters (a–d) in the same column are significantly different. Means with different capital letters (A–D) in the same row are significantly different. Number of replicate = 3. Abbreviations: DPPH, 2,2-diphenyl-1-picrylhydrazyl; FRAP, ferric reducing antioxidant power.

**Table 3:** Quantification of some targeted compounds in samples obtained from different solvent extraction systems.

Plant Sources	Yield of Sample Extract and Quantity of Active Compounds in Different Solvent Extraction			
	Acetone	Methanol	Ethanol	Water
**Turmeric**				
Yield (%)	17.6 ± 1.2 ^A,^**	8.3 ± 0.9 ^C^	10.6 ± 1.1 ^B^	3.2 ± 0.2 ^D^
Curcumin *	510.8 ± 0.2 ^A^	119.4 ± 0.3 ^C^	280.9 ± 0.3 ^B^	0.2 ± 0.1 ^D^
Desmethoxycurcumin	133.1 ± 0.1 ^A^	32.2 ± 0.0 ^C^	81.1 ± 0.1 ^B^	0.1 ± 0.0 ^D^
Bisdesmethoxycurcumin	107.2 ± 0.4 ^A^	29.0 ± 0.4 ^C^	69.0 ± 1.0 ^B^	0.1 ± 0.0 ^D^
**Curry leaf**				
Yield (%)	10.8 ± 0.8 ^B^	13.3 ± 1.1 ^A^	12.5 ± 0.9 ^A^	6.6 ± 0.6 ^C^
Rutin	0.8 ± 0.0 ^B^	0.7 ± 0.0 ^C^	0.9 ± 0.0 ^A^	0.1 ± 0.0 ^D^
Quercetin-3-glycoside	4.2 ± 0.0 ^C^	4.8 ± 0.0 ^B^	5.4 ± 0.0 ^A^	1.0 ± 0.1 ^D^
Myrecitin	1.8 ± 0.0 ^B^	2.2 ± 0.0 ^A^	2.4 ± 0.0 ^A^	0.1 ± 0.0 ^C^
Quercetin	1.0 ± 0.1 ^B^	0.9 ± 0.0 ^C^	1.4 ± 0.1 ^A^	0.1 ± 0.0 ^D^
**Torch ginger**				
Yield (%)	12.3 ± 0.8 ^B^	12.9 ± 1.4 ^B^	17.2 ± 1.9 ^A^	11.8 ± 0.8 ^B,C^
Chlorogenic acid	11.5 ± 0.1 ^D^	18.7 ± 0.1 ^B^	21.8 ± 0.3 ^A^	13.4 ± 0.6 ^C^
**Lemon grass**				
Yield (%)	11.9 ± 1.0 ^A^	10.2 ± 0.1 ^B^	9.6 ± 0.5 ^C^	5.1 ± 0.9 ^C^
Caffeic acid	0.3 ± 0.0 ^A^	0.1 ± 0.0 ^B^	0.1 ± 0.0 ^C^	0.1 ± 0.0 ^D^
*p*-coumaric acid	0.5 ± 0.0 ^A^	0.3 ± 0.0 ^B^	0.3 ± 0.0 ^C^	0.0
luteolin-7-*o*-glycoside	0.6 ± 0.1 ^A^	0.4 ± 0.0 ^B^	0.4 ± 0.0 ^C^	0.2 ± 0.0 ^D^

* Concentrations of all phytochemicals were reported based on mg/g of the freeze-dried crude extract; ** (A–D) Means within the same row with different letters are significantly different; Number of replicates = 3.
